# Tree Shrew as an Emerging Model for Aging Research: Characterization of Senescence and Therapeutic Interventions

**DOI:** 10.1111/acel.70781

**Published:** 2026-11-03

**Authors:** Qiuni Luo, Wenxian Xiao, Xin Dong, Ayesha Nisar, Yuhua Ma, Xiang Lai, Raoxian Bai, Meiting Zi, Qi Geng, Qingyu Zhang, Cheng Zhang, Qiong Wang, Naheemat Modupeola Gold, Zhen Zhou, Li Hu, Ao Zhang, Qinghua Zeng, Jing Liu, Sawar Khan, Longbao Lv, Yonghan He

**Affiliations:** ^1^ State Key Laboratory of Genetic Evolution & Animal Models, Key Laboratory of Healthy Aging Research of Yunnan Province Kunming Institute of Zoology, Chinese Academy of Sciences Kunming Yunnan China; ^2^ University of Chinese Academy of Sciences Beijing China; ^3^ National Resource Center for Non‐Human Primates, and National Research Facility for Phenotypic & Genetic Analysis of Model Animals (Primate Facility) Kunming Institute of Zoology, Chinese Academy of Sciences Kunming Yunnan China; ^4^ School of Chemical Science and Technology Yunnan University Kunming Yunnan China; ^5^ Laboratory of Molecular Genetics of Aging and Tumor, Medical School Kunming University of Science and Technology Kunming Yunnan China; ^6^ Key Laboratory of Biodiversity Information Kunming Institute of Zoology, Chinese Academy of Sciences Kunming Yunnan China; ^7^ KIZ/CUHK Joint Laboratory of Bioresources and Molecular Research in Common Diseases Kunming Yunnan China

**Keywords:** aging model, cellular senescence, senescence‐associated secretory phenotype, senolytic, tree shrew

## Abstract

Aging is characterized by progressive physiological decline and increased susceptibility to age‐related diseases. Conventional rodent models have contributed significantly to our understanding of aging, yet their translational relevance is limited by substantial evolutionary divergence from humans. Here, we introduce the tree shrew ( *Tupaia belangeri chinensis* ) as a novel and biologically relevant model organism for aging research. In vitro, we induced cellular senescence in tree shrew fibroblasts through ionizing radiation, doxorubicin treatment, or replicative exhaustion, all of which consistently triggered robust senescent phenotypes and a pronounced senescence‐associated secretory phenotype (SASP), accompanied by upregulation of conserved senescence biomarkers. In vivo, aged tree shrews exhibited progressive physical frailty, metabolic dysfunction, organ fibroplasia, and spontaneous tumorigenesis. Transcriptomic profiling uncovered widespread age‐related alterations across multiple tissues, prominently affecting pathways involved in chronic inflammation, extracellular matrix remodeling, and metabolic dysregulation. Further comparative transcriptomic analyses of liver and spleen tissues confirmed that tree shrews recapitulate conserved human aging transcriptional trajectories and core gene expression patterns, whereas mice displayed markedly divergent aging signatures. Notably, senolytic intervention with dasatinib and quercetin (D + Q) effectively reversed key transcriptional changes and ameliorated selected age‐related pathologies, including hepatic steatosis. Importantly, the D + Q‐induced transcriptomic responses in tree shrews exhibited markedly greater concordance with human aging signatures than did corresponding mouse datasets. Collectively, these findings establish the tree shrew as a translationally relevant aging model that bridges the gap between fundamental discovery and clinical application.

## Graphical abstract

Compared with mouse cells, tree shrew fibroblasts more faithfully recapitulate the hallmarks of human cellular senescence. In vivo, aged tree shrews exhibit transcriptomic signatures of aging across multiple organs that closely mirror those observed in humans. Notably, senolytic D + Q treatment partially reverses these age‐related molecular and tissue phenotypes, eliciting a more robust transcriptional response in tree shrews than in mice. The graphical abstract was created with BioRender.com.

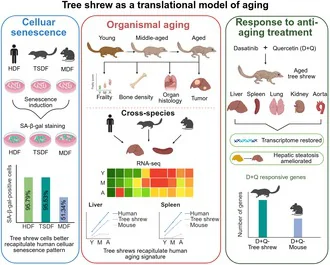

## 1 Introduction

Aging is a complex, multifactorial biological process that gradually impairs cellular, tissue, and organ function, ultimately increasing the risk of chronic diseases such as cancer, cardiovascular disease, neurodegeneration, and metabolic dysfunction (Nisar et al. 2024). At the cellular level, aging is characterized by hallmarks such as genomic instability, epigenetic alterations, telomere attrition, loss of proteostasis, and cellular senescence (López‐Otín et al. 2023). Among these, cellular senescence is a stable state of proliferative arrest accompanied by the secretion of inflammatory and tissue‐remodeling factors collectively termed the senescence‐associated secretory phenotype (SASP), which has emerged as a key driver of tissue dysfunction and age‐related pathology (Calcinotto et al. 2019; Saito et al. 2024). Targeting cellular senescence with senotherapy has shown promise in extending healthspan and mitigating diseases in preclinical models, thereby fueling interest in identifying more representative and translational animal models for aging research (Calcinotto et al. 2019; Hickson et al. 2019; Samarawickrama et al. 2024).

Rodent models, particularly the laboratory mouse ( *Mus musculus* ), have historically served as the cornerstone of aging research due to their short lifespan, genetic manipulability, and well‐established biological tools (Ackert‐Bicknell et al. 2015; Vanhooren and Libert 2013). However, rodents are evolutionarily distant from humans and often fail to fully recapitulate the complexity of human aging, particularly in immune, metabolic, and neurological systems (Ma et al. 2022). For instance, elevated glucose levels have been correlated with decreased mortality in mice, whereas in nonhuman primates (NHPs) and humans, higher glucose is associated with increased mortality (Palliyaguru et al. 2021). Certain inflammatory responses, immune responses, and tissue regenerative capacities differ substantially between rodents and primates, leading to limitations in translating findings to clinical applications (Seok et al. 2013; Wei et al. 2025). Although NHPs provide closer genetic and physiological relevance, they are constrained by ethical concerns, high maintenance costs, long lifespans, and limited accessibility (Nadon 2006).

The tree shrew ( *Tupaia belangeri chinensis* ) has recently gained attention as a promising intermediate model organism (Yao et al. 2024). Belonging to the order Scandentia, tree shrews are phylogenetically closer to primates than rodents, offering an evolutionary bridge between traditional rodent models and NHPs (Fan et al. 2013). Tree shrews share high genomic similarity with humans, display complex behaviors, and possess physiological systems that more closely resemble those of primates. They are small, cost‐effective, breed prolifically, and are subject to less stringent ethical constraints compared with NHPs (Shen et al. 2011; Yao et al. 2024). Moreover, tree shrews are increasingly used in biomedical research areas, including neuroscience, infectious disease, and cancer biology (Xu et al. 2013), yet their potential for aging‐related research remains largely underexplored.

Given the limitations of existing models and the urgent need for more predictive and scalable systems, we aimed to evaluate and establish the tree shrew as a suitable model for aging and age‐related phenotype research. We hypothesized that the tree shrew would exhibit canonical features of aging at the cellular, physiological, and molecular levels, and that these features would be modifiable by senolytic treatment. To this end, we performed a comprehensive characterization of aging in tree shrews using both in vitro and in vivo approaches. Our findings demonstrate that the tree shrew faithfully models key features of mammalian aging, including multi‐organ senescence, age‐related degeneration, and responsiveness to senolytics. The model offers both evolutionary relevance and practical feasibility, enabling larger‐scale aging studies that are currently not feasible in NHPs. Collectively, this work supports the tree shrew as a novel and valuable platform for translational aging research and the preclinical evaluation of anti‐aging interventions.

## 2 Results

### 2.1 In Vitro Senescence Modeling in Tree Shrew Fibroblasts

Phylogenetic analysis has indicated that the tree shrew exhibits closer evolutionary relationships to primates (including humans and macaques) than to rodents (such as mice and rats) (Figure 1A) (Fan et al. 2019; Ye et al. 2021). This evolutionary proximity is a crucial advantage, offering a genetic and physiological context more comparable to humans while maintaining the practical benefits of a small mammalian model. Tree shrew dentition and claw morphology show greater similarity to those of rhesus macaques ( *Macaca mulatta* ) than rodents such as mice (Figure S1A,B), suggesting phylogenetic proximity in functional traits. In addition to morphological traits, the histological structure of tree shrew skin closely resembles that of humans and NHPs (Zhang, Luo, et al. 2020). To further assess its relevance in aging studies, we developed and validated an in vitro senescence model using tree shrew dermal fibroblasts (TSDF), which were isolated from skin tissues (Figure 1B,C).

**FIGURE 1:**
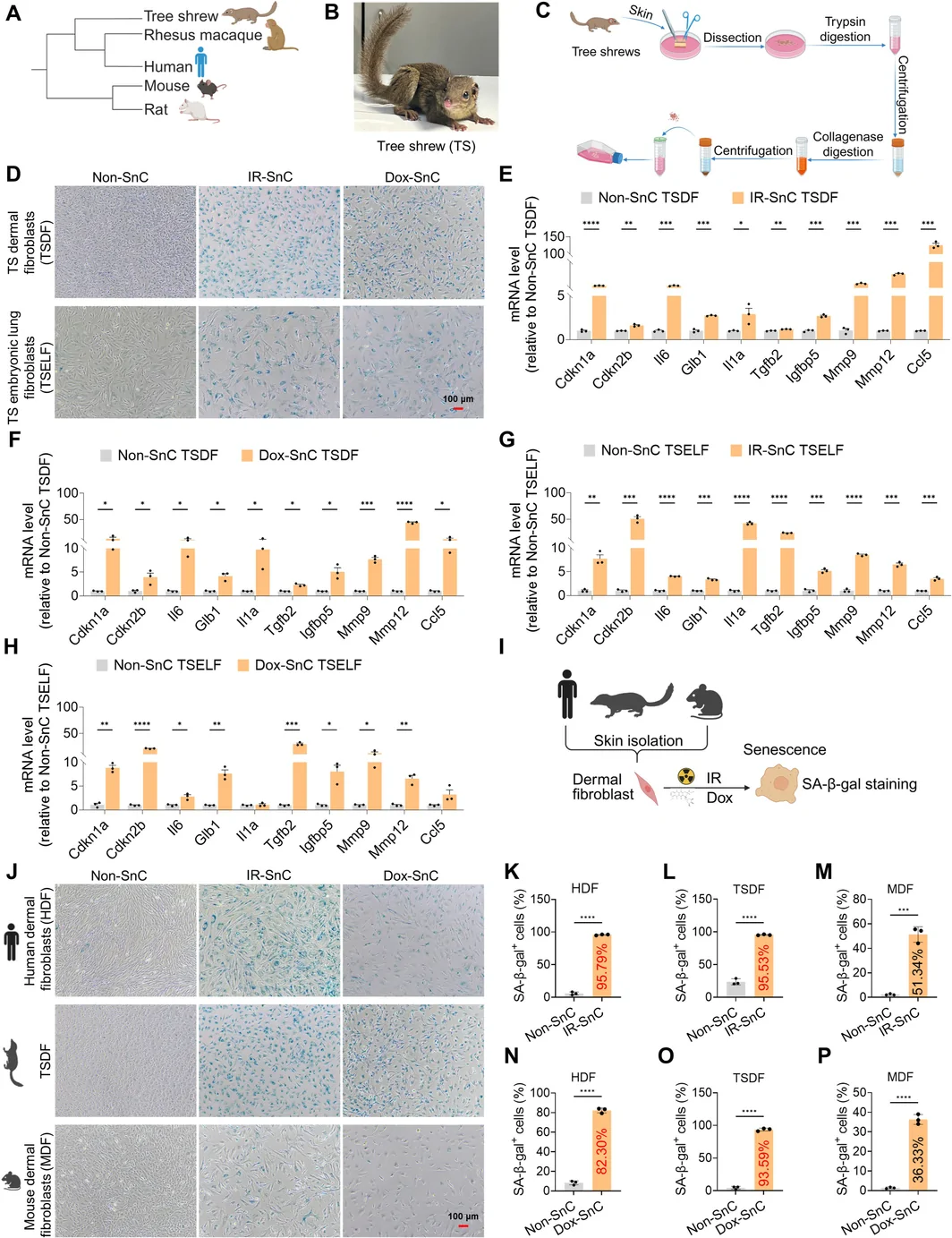
Establishment and characterization of senescence models in tree shrew fibroblasts. (A) Phylogenetic relationship among tree shrews, rhesus macaques, humans, mice, and rats, highlighting the evolutionary proximity of tree shrews to primates. (B) Representative image of a tree shrew (TS), the animal source for fibroblast isolation. (C) Schematic workflow for isolation of dermal fibroblasts from tree shrew skin. (D) SA‐β‐gal staining of nonsenescent cells (non‐SnC), ionizing radiation‐induced senescent cells (IR‐SnC), and doxorubicin‐induced senescent cells (Dox‐SnC) in tree shrew dermal fibroblasts (TSDF, top) and embryonic lung fibroblasts (TSELF, bottom). Blue‐stained cells mark senescent cells. (E–H) qRT‐PCR analysis of canonical senescence‐associated genes (*Cdkn1a*, *Cdkn2b*, *Il6*, *Glb1*, *Il1a*, *Tgfb2*, *Igfbp5*, *Mmp9*, *Mmp12*, *Ccl5*). (E) IR‐SnC versus non‐SnC TSDF; (F) Dox‐SnC versus non‐SnC TSDF; (G) IR‐SnC versus non‐SnC TSELF; (H) Dox‐SnC versus non‐SnC TSELF. Data are mean ± SEM (*n* = 3 biological replicates). (I) Experimental design for inducing cellular senescence in human, tree shrew, and mouse dermal fibroblasts via IR or Dox treatment, followed by SA‐β‐gal staining. (J) Representative SA‐β‐gal staining images of nonsenescent, IR‐induced senescent, and Dox‐induced senescent dermal fibroblasts from human (HDF), tree shrew (TSDF), and mouse (MDF). Scale bar, 100 μm. (K–M) Quantification of the percentage of SA‐β‐gal‐positive cells in IR‐induced senescent HDF, TSDF, and MDF relative to non‐SnC controls. (N–P) Quantification of the percentage of SA‐β‐gal‐positive cells in Dox‐induced senescent HDF, TSDF, and MDF relative to non‐SnC controls. Data are represented as mean ± SEM. Statistical significance was assessed using an unpaired two‐tailed Student's *t*‐test. *p < 0.05, **p < 0.01, ***p < 0.001, ****p < 0.0001; *p*‐values correspond to the indicated comparisons. Figure 1A,C,I were created with BioRender.com.

Cellular senescence in TSDF was induced via two classical experimental approaches: ionizing radiation (IR) and doxorubicin (Dox), as previously described (Hu et al. 2023). Cells exposed to these treatments displayed canonical hallmarks of senescence, including flattened morphology, growth arrest, and positive staining for senescence‐associated β‐galactosidase (SA‐β‐gal) (Figure 1D). At the molecular level, quantitative real‐time PCR (qRT‐PCR) validation confirmed significant upregulation of core senescence markers and SASP genes in IR‐ and Dox‐induced senescent TSDF, including *Cdkn1a*, *Cdkn2b*, *Il6*, *Glb1*, *Il1a*, *Tgfb2*, *Igfbp5*, *Mmp9*, *Mmp12*, and *Ccl5* (Figure 1E,F). In addition, replicative senescence induced by serial passaging also triggered increased SA‐β‐gal staining and elevated expression of SASP transcripts in TSDF (Figure S1C–E). Comparable senescence phenotypes were also validated in tree shrew embryonic lung fibroblasts (TSELF) (Figure 1D,G,H).

Given that SA‐β‐gal activity is widely regarded as a well‐established hallmark marker of cellular senescence (Debacq‐Chainiaux et al. 2009), we further compared SA‐β‐gal activity in dermal fibroblasts across human, tree shrew, and mouse to explore species differences in senescence patterns. Under identical IR treatment conditions, both human dermal fibroblasts (HDF) and TSDF exhibited a high proportion of SA‐β‐gal‐positive cells (95.79% for HDF and 95.53% for TSDF), whereas only 51.34% of mouse dermal fibroblasts (MDF) were SA‐β‐gal‐positive under the same conditions (Figure 1I–M). A similar pattern was observed following Dox treatment, with 82.30% of HDF and 93.59% of TSDF being SA‐β‐gal‐positive, compared with only 36.33% of MDF (Figure 1J,N–P). These results indicate that tree shrew cells may better recapitulate the senescence pattern of human cells.

To further explore transcriptomic changes, we subjected IR‐induced HDF and TSDF to RNA sequencing (RNA‐seq). IR‐induced senescent HDF and TSDF shared 282 upregulated and 106 downregulated genes (Figure S1F,G). Gene Set Variation Analysis (GSVA) revealed that TSDF exhibited senescence‐associated changes highly similar to those in HDF, including cell cycle inhibition (Mdm2‐p21 regulation), epigenetic alterations (cytosine and DNA methylation, PRC2 pathway, chromatin organization), loss of protein homeostasis (ribosome, translation initiation, and elongation), metabolic dysregulation (IRS activation, insulin signaling, cholesterol biosynthesis, mevalonate pathway, and β‐oxidation), and pathway changes commonly observed in senescence, such as TP53 and FOXO activity regulation (Figure S1H). Together, these findings demonstrate that tree shrew fibroblasts respond to senescence‐inducing stimuli in a manner highly analogous to human cells, supporting their utility as a relevant model for mechanistic aging studies.

### 2.2 Age‐Related Physiological and Histopathological Changes in Tree Shrews

To extend our findings to in vivo aging, we performed comprehensive physiological, biochemical, and histological analyses across three age groups of tree shrews: young (1–2 years), middle‐aged (3–4 years), and aged (6–8 years) (Figure 2A). We first conducted a clinical frailty score assessment to evaluate overall health status across age groups, examining body condition, alopecia, thermoregulation, jumping ability, and dental and eye health. The results demonstrated progressive accumulation of health deficits with advancing age (Figure 2B–G; Figure S2A). These frailty patterns closely mirror the human clinical frailty index, indicative of systemic functional decline (Whitehead et al. 2014).

**FIGURE 2:**
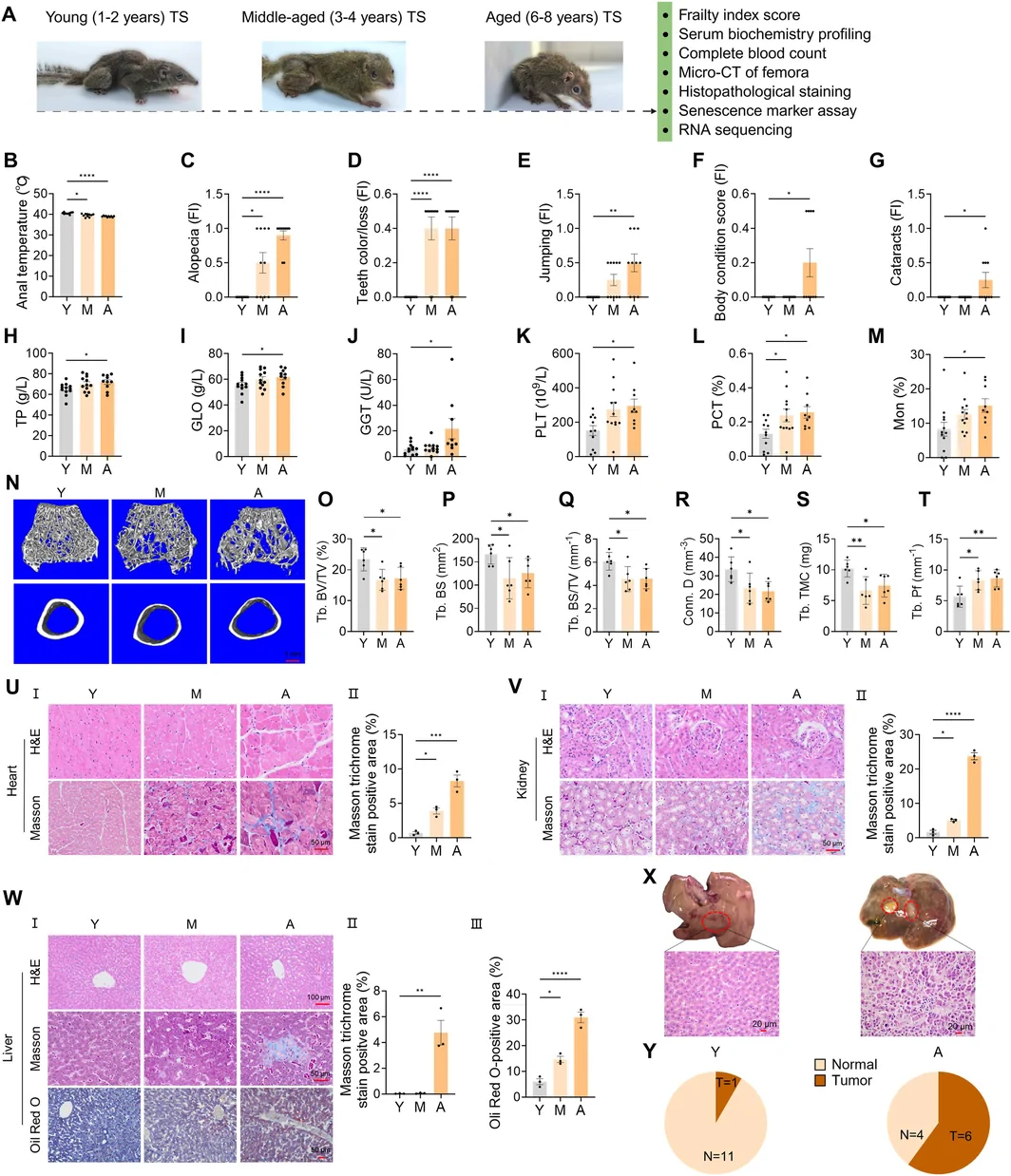
Age‐related physiological, biochemical, bone, and histopathological changes in tree shrews. (A) Representative images of tree shrews categorized by age: Young (1–2 years), middle‐aged (3–4 years), and aged (6–8 years). (B–G) Frailty index assessments of physiological parameters across age groups (Y = young, M = middle‐aged, A = aged), including anal temperature (B), alopecia score (C), teeth color/loss (D), jumping ability (E), body condition score (F), and cataract formation (G). (H–M) Blood biochemical and hematological parameters measured across age groups: (H) total protein, TP; (I) globulin, GLO; (J) γ‐glutamyl transferase, GGT; (K) platelet count, PLT; (L) plateletcrit, PCT%; and (M) percentage of monocytes, Mon%. (N) Representative 3D reconstructed micro‐CT images of the distal femoral metaphysis from young (Y), middle‐aged (M), and aged (A) tree shrews. Scale bar, 1 mm. (O–T) Quantitative micro‐CT analysis of trabecular bone microarchitecture (*n* = 6 per group for all panels): (O) trabecular bone volume fraction (Tb. BV/TV); (P) trabecular bone surface (Tb. BS); (Q) trabecular bone surface‐to‐volume ratio (Tb. BS/TV); (R) connectivity density (Conn. D); (S) trabecular mineral content (Tb. TMC); (T) trabecular pattern factor (Tb. Pf). (U) Representative H&E and Masson's trichrome staining (left, I) and quantitative analysis of Masson's trichrome staining (right, II) of heart tissues from young (Y), middle‐aged (M), and aged (A) tree shrews. Scale bar, 50 μm. (V) Representative H&E and Masson's trichrome staining (left, I) and quantitative analysis of Masson's trichrome staining (right, II) of kidney tissues from young (Y), middle‐aged (M), and aged (A) tree shrews. Scale bar, 50 μm. (W) Representative H&E, Masson's trichrome, and Oil Red O staining (left, I) of liver across age groups, together with quantitative analysis of collagen deposition (middle, II) and lipid accumulation (right, III). Scale bars: H&E, 100 μm; Masson's trichrome, 50 μm; Oil Red O, 50 μm. (X) Gross pathology and H&E staining of normal (young) versus tumor‐bearing (aged) livers. H&E staining of aged liver shows hepatic tumor incidence (dashed circle). Scale bar, 20 μm. (Y) Quantification of liver tumor incidence; 1 out of 12 young animals and 6 out of 10 aged animals developed liver tumors. The data presented are mean ± SEM. *p < 0.05, **p < 0.01, ***p < 0.001, and ****p < 0.0001 for the indicated comparisons. Figure 2A was created with BioRender.com.

Biochemical profiling revealed that aged tree shrews displayed elevated serum total protein (TP), globulin (GLO), and γ‐glutamyl transferase (GGT) levels, suggesting age‐related hepatic and immune alterations (Figure 2H–J) (Dong et al. 2024; Zeng et al. 2024). Concurrent changes in hematological indices, including platelet count (PLT), plateletcrit (PCT%), and percentage of monocytes (Mon%)—further indicated hematopoietic and vascular aging (Figure 2K–M). No statistically significant differences were observed among the age groups in other biochemical and hematological parameters (Figure S2B–M).

We next characterized skeletal aging phenotypes using micro‐CT. Middle‐aged and aged tree shrews exhibited marked reductions in trabecular bone volume fraction (Tb. BV/TV), trabecular bone surface (Tb. BS), trabecular bone surface‐to‐volume ratio (Tb. BS/TV), connectivity density (Conn. D), and trabecular mineral content (Tb. TMC). In contrast, trabecular pattern factor (Tb. Pf) was increased (Figure 2N–T). Histopathological assessments using hematoxylin and eosin (H&E) and Masson's trichrome staining revealed substantial age‐associated tissue remodeling. In the heart, we observed cardiomyocyte hypertrophy and a significant increase in fibrosis (Figure 2U) (Hu et al. 2022; Tamiato et al. 2024). Aged kidney sections displayed glomerular atrophy, collagen deposition, calcification, lipofuscin accumulation, and markedly increased fibrosis (Figure 2V; Figure S2N) (Wu et al. 2024). Aged liver tissues exhibited prominent fibroplasia and steatotic changes (Figure 2W). Notably, aged animals showed a higher incidence of spontaneous hepatic tumors (6/10) compared with young animals (1/12), suggesting that tree shrews can model age‐related oncogenesis (Figure 2X,Y). Histological analyses of additional organs also revealed systemic aging features. For example, middle‐aged and aged tree shrews exhibited enlarged alveolar spaces and reduced alveolar cell density in the lung (Figure S2O). The spleen showed reduced follicle size and decreased number of lymphoid nodules (Figure S2P). Additionally, brown adipose tissues showed hypertrophic fat cells; ovarian sections revealed diminished primordial follicle reserves; and quadriceps muscle cells showed nuclear displacement and a decrease in cell size (Figure S2Q–S). Collectively, these multi‐system pathological phenotypes demonstrate that tree shrews recapitulate key hallmarks of mammalian organismal aging and support their utility for multi‐organ aging research.

### 2.3 Systemic Increase in SASP Gene Expression With Age Across Tissues of the Tree Shrew

The SASP represents a pro‐inflammatory secretory program triggered within senescent cells, which promotes tissue dysfunction and fuels organismal aging. To characterize organism‐wide SASP activation during aging, we quantified transcript levels of core senescence markers (*Cdkn1a*, *Cdkn2b*) and a panel of SASP‐related genes across seven tree shrew tissues, including the heart, kidney, liver, spleen, lung, aorta, and brain. We detected heterogeneous upregulation of senescence‐associated genes across organs, revealing both conserved and tissue‐restricted SASP signatures. In aged heart tissue, *Cdkn1a*, *Cdkn2b*, *Il1b*, and *Mmp9* were significantly induced (Figure 3A). The kidney displayed elevated transcripts of *Cdkn1a*, *Igfbp2*, *Tgfb2*, *Cxcl10*, *Il1b*, and *Mmp9* (Figure 3B). In the liver, *Cdkn1a*, *Cdkn2b*, *Igfbp2*, and *Mmp12* showed prominent age‐dependent upregulation (Figure 3C). The spleen exhibited increased expression of *Il6*, *Il1a*, and *Mmp9* (Figure 3D). In lung tissue, *Il1a*, *Mmp12*, and *Mmp9* were markedly upregulated with age (Figure 3E). The aorta showed robust induction of *Il1a*, *Cxcl10*, *Mmp12*, *Il1b*, and *Mmp9* (Figure 3F), while the brain presented elevated *Cdkn2b*, *Il6*, and *Mmp9* transcripts in aged animals (Figure 3G). These elevations were particularly prominent in metabolically active organs such as the liver and kidney, as well as in vasculature (aorta) and heart, where inflammation and remodeling are key aging features. To visualize the overlap and tissue specificity of SASP activation, we constructed an UpSet plot (Figure 3H). This analysis revealed both shared and unique gene expression patterns across organs. For instance, *Mmp9* was commonly upregulated in multiple tissues, whereas other genes like *Tgfb2* showed more restricted expression patterns. This mosaic SASP expression profile suggests both systemic and organ‐specific aspects of senescence in tree shrews and offers a platform for dissecting tissue‐selective aging mechanisms.

**FIGURE 3:**
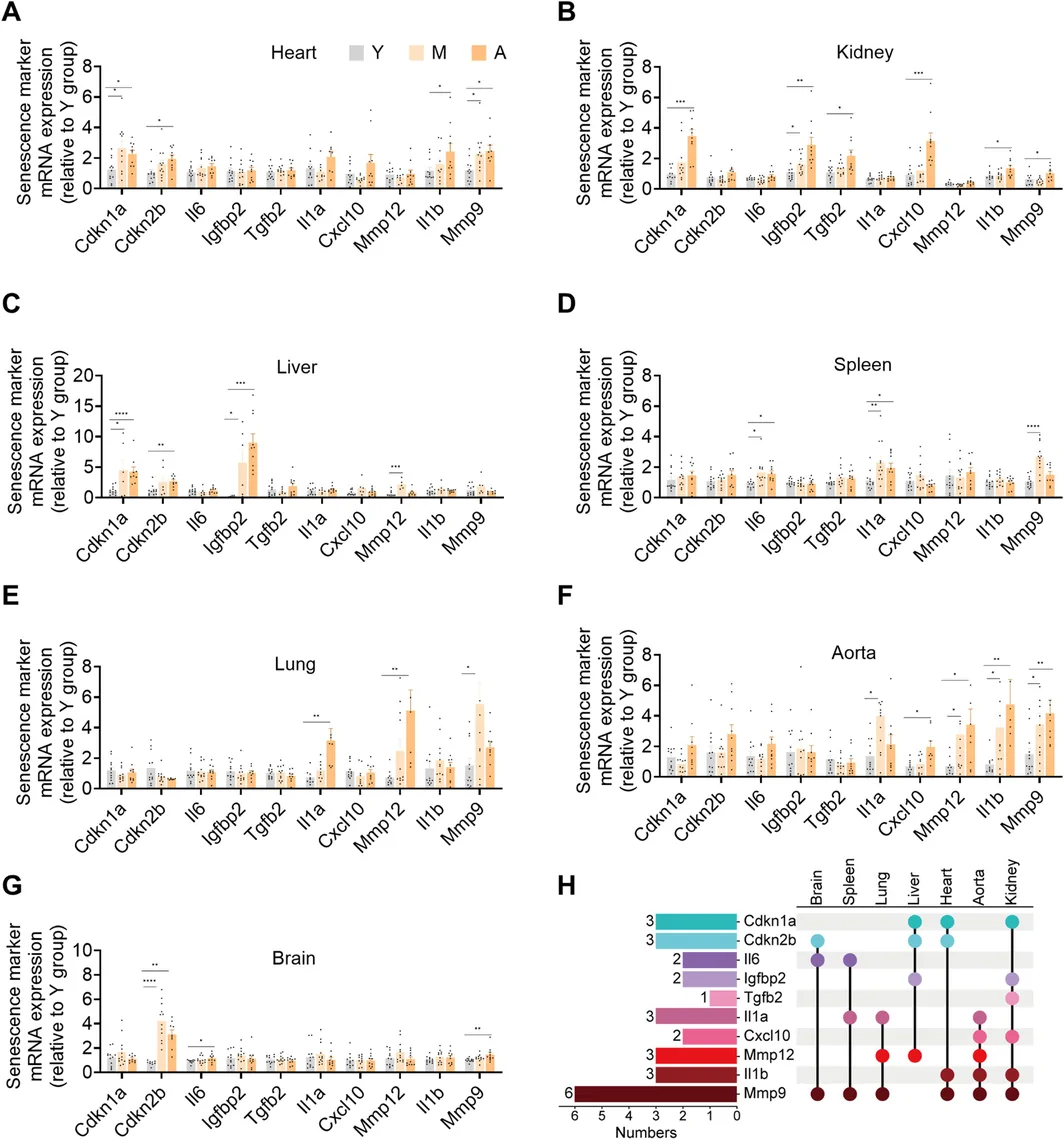
Age‐related mRNA expression of SASP factors across tissues in tree shrews. mRNA expression levels of key senescence markers and senescence‐associated secretory phenotype (SASP) factors, including *Cdkn1a*, *Cdkn2b*, *Il6*, *Igfbp2*, *Tgfb2*, *Il1a*, *Cxcl10*, *Mmp12*, *Il1b*, and *Mmp9*, were profiled across multiple tissues from young (Y, gray), middle‐aged (M, beige), and aged (A, orange) tree shrews. Analyzed tissues: (A) heart; (B) kidney; (C) liver; (D) spleen; (E) lung; (F) aorta; and (G) brain. (H) UpSet plot showing overlaps of significantly up‐regulated SASP‐related genes across tissues upon comparison of middle‐aged versus young and aged versus young animals. Data are presented as mean ± SEM. Statistical significance was assessed using one‐way ANOVA with Tukey's multiple‐comparisons test; *p < 0.05, **p < 0.01, ***p < 0.001, and ****p < 0.0001.

### 2.4 Age‐Dependent Transcriptomic Profiles in the Liver and Spleen Reveal Age‐Associated Dysfunction

Given the critical roles of the liver and spleen in systemic homeostasis and age‐related changes in histology (Figure 2W,X; Figure S2P), we performed transcriptomic profiling to dissect molecular signatures of aging in these two organs. Weighted Gene Co‐Expression Network Analysis (WGCNA) identified 20 co‐expression modules in the liver (Figure 4A; Figure S3A). Among these modules, the “purple” module displayed the strongest positive correlation with chronological age (Figure 4B,C). In parallel, Mfuzz (model‐based fuzzy clustering for time series data) partitioned hepatic genes into four age‐dependent expression trajectories (Figure 4D). Genes within Cluster 1 were progressively upregulated with age, whereas Cluster 2 genes were downregulated over time. Cluster 3 exhibited an up‐then‐down pattern, and Cluster 4 followed a down‐then‐up trajectory. We integrated genes derived from the age‐correlated purple module and age‐upregulated Cluster 1 and obtained 46 overlapping consensus genes (Figure 4E). Gene Ontology (GO) enrichment analysis of these 46 genes uncovered three major functional categories (Figure 4F): (1) Lipid metabolic reprogramming (lipid droplet, phospholipid biosynthetic process, regulation of fat cell differentiation); (2) Inflammation and oxidative stress response (cytokine activity, sulfur compound binding), and (3) Tissue microenvironment remodeling (cell adhesion mediator activity, angiogenesis, enzyme‐linked receptor signaling). Specifically, we observed age‐dependent upregulation of several canonical and emerging senescence‐associated genes, including Adipogenesis regulatory factor (*Adirf*), Aquaporin 7 (*Aqp7*), Armadillo repeat containing X‐linked 4 (*Armcx4*), B‐cell lymphoma 6B (*Bcl6b*), Heme‐binding protein 2 (*Hebp2*), and Polymeric immunoglobulin receptor (*Pigr*) (Figure 4G).

**FIGURE 4:**
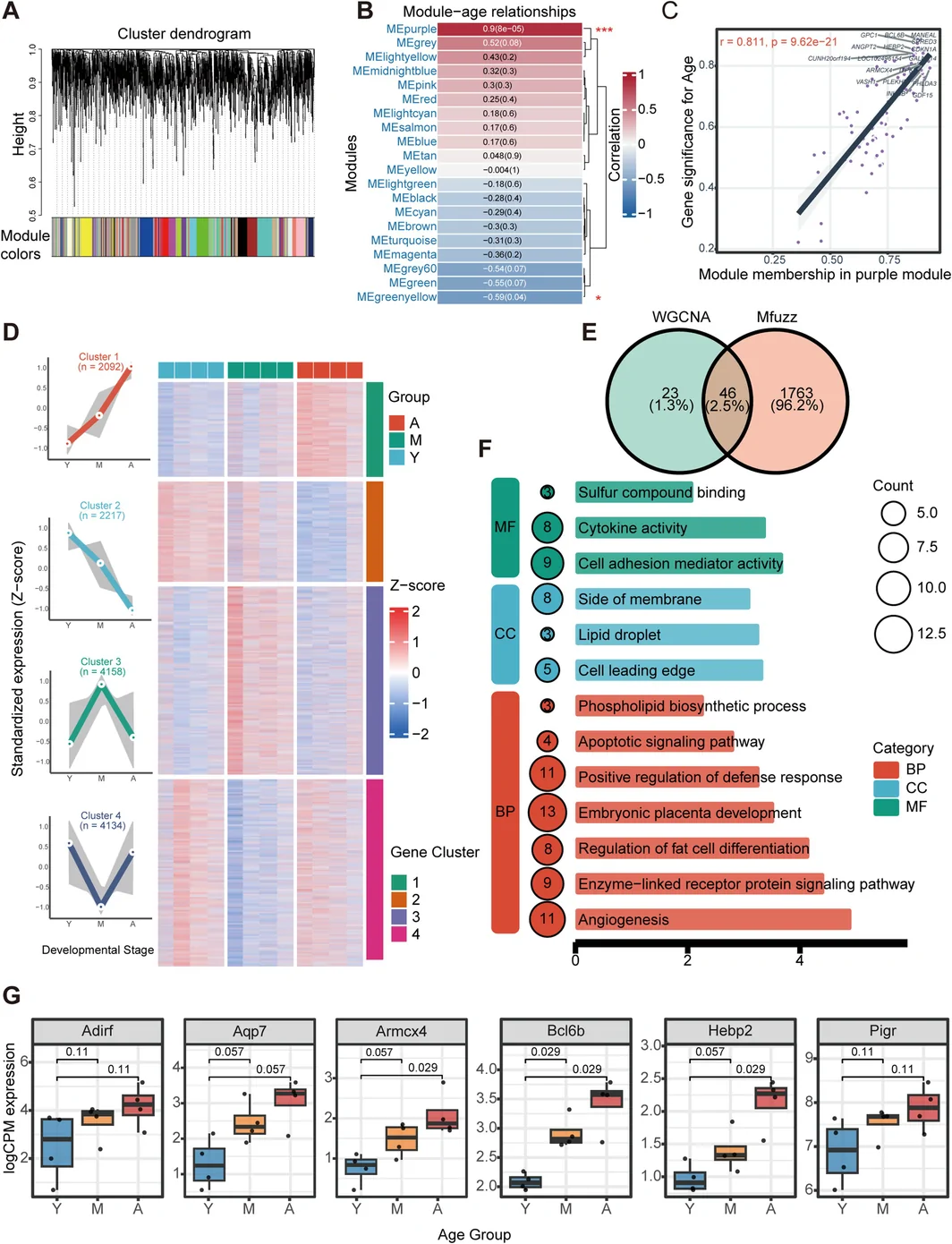
Hepatic pathway alterations in aged tree shrews. (A) Hierarchical clustering dendrogram of co‐expressed genes from liver transcriptomes; genes were partitioned into 20 distinct modules using weighted gene co‐expression network analysis (WGCNA). (B) Module‐trait relationships displaying Pearson correlation coefficients between module eigengenes and chronological age. (C) Scatterplot of module significance (x‐axis) versus gene significance (y‐axis) for the age‐correlated purple module. (D) Tree shrew liver genes grouped into four distinct expression trends by Mfuzz soft‐clustering analysis. (E) Venn diagram showing the overlap between age‐upregulated genes (Cluster 1) and genes within the purple WGCNA module. (F) GO enrichment network for overlapping genes, highlighting biological processes associated with cellular senescence and tissue aging. (G) Bar plots showing age‐dependent mRNA expression levels of six senescence‐associated genes in tree shrews.

We subsequently applied the identical WGCNA‐Mfuzz analytical workflow to splenic transcriptomic datasets and identified a “red” gene module that exhibited a significant inverse correlation with chronological age (Figure S3B–E). Mfuzz clustering also resolved four primary age‐associated gene‐expression trajectories for splenic genes (Figure S3F), among which Cluster 2 genes were consistently downregulated with advancing age. By intersecting genes within the “red” module and Cluster 2, we identified 37 consensus genes jointly downregulated during aging (Figure S3G). GO enrichment analysis of these 37 genes highlighted two major functional deficits in aged spleen (Figure S3H): (1) Immune impairment, represented by pathways such as carbohydrate binding, receptor complex, response to lipopolysaccharide, and organic anion transport; (2) Neuroimmune dysregulation, involving regulation of cell‐substrate adhesion, neuron projection development, and striated muscle cell differentiation.

To further uncover evolutionarily conserved molecular signatures of aging, we used mouse datasets from Tabula Muris Senis (Schaum et al. 2019) and human datasets from GTEx, and compared age‐dependent transcriptomic profiles in the liver and spleen across humans, tree shrews, and mice. We focused on the liver and spleen because the liver is the primary metabolic organ responsible for maintaining systemic metabolic homeostasis, while the spleen is the largest lymphoid organ mediating immune surveillance (Lin et al. 2023; Tarantino et al. 2013). Mfuzz clustering resolved four predominant age‐associated gene expression trajectories in the liver of all three species, with human age‐related genes mainly distributed in the Young (3254 genes) and Aged (2863 genes) trajectories. Cross‐species comparison of homologous genes within each trajectory revealed distinct conservation patterns. In the Aged trajectory, 270 of the 2863 human genes were shared with mouse and 208 with tree shrew, whereas the majority (2423) were human‐specific; in the Young trajectory, 475 human genes overlapped with mouse and 353 with tree shrew. The Middle‐trough trajectory was highly conserved with both species (641 with mouse, 494 with tree shrew). Notably, in the inverted V‐shaped Middle‐peak trajectory, 630 human genes (24.1%) overlapped with tree shrew orthologs but only 175 (6.7%) with mouse, of which 576 were shared with tree shrews but not mice, indicating that tree shrews recapitulated human‐specific mid‐life transcriptional activation during aging (Figure S4A,B). We next examined representative hepatic aging‐related genes, including *Hmox1* (heme oxygenase 1) (Sato et al. 2022), *Prdx1* (peroxiredoxin 1) (Zhang, Li, et al. 2020), *Lonp1* (lon peptidase 1, mitochondrial) (Chimienti et al. 2021), *Prf1* (perforin 1) (Gong et al. 2019), D*dit3* (DNA‐damage‐inducible transcript 3) (Yong et al. 2021), *Atg14* (autophagy‐related 14), *Mdm2* (MDM2 proto‐oncogene) (Wu and Prives 2018), and *Prkaa1* (protein kinase AMP‐activated catalytic subunit alpha 1) (Zheng et al. 2018). Notably, *Hmox1* and *Prkaa1* displayed opposite age‐dependent expression trends in mice relative to humans and tree shrews, whereas *Prf1* showed no significant age‐associated alterations. In addition, *Ddit3*, *Atg14*, and *Mdm2* exhibited divergent age‐related expression patterns in mice compared with humans and tree shrews (Figure S4C,D).

Consistent with our hepatic observations, splenic transcriptome analysis revealed highly conserved aging trajectory patterns across the three species but with distinct gene set sizes. Human age‐related genes were more evenly distributed across trajectories, with Young and Aged containing 4251 and 3523 genes, respectively. The Young trajectory was predominantly conserved with mouse: 1776 of the 4251 human Young genes (41.8%) overlapped with mouse orthologs, compared with 516 with tree shrew. By contrast, in the Aged trajectory, 454 human genes (12.9%) were shared with tree shrew versus only 112 (3.2%) with mouse, of which 437 were exclusive to tree shrew. In the V‐shaped Middle‐trough module, overlap with tree shrew (415) again exceeded that with mouse (148), while both humans (3135) and tree shrews (3168) possessed markedly more genes than mice (1076); the age‐upregulated gene sets in humans (3523) and tree shrews (2885) were also considerably larger than those in mice (634). The Middle‐peak trajectory showed comparable overlap with both species (306 with tree shrew, 276 with mouse) (Figure S5A,B). We further found representative age‐repressed splenic genes, including *Pcna* (proliferating cell nuclear antigen) (Wang et al. 2020), *Gclm* (glutamate‐cysteine ligase modifier subunit) (Zhou et al. 2021), *Txn* (thioredoxin), and *Serpine2* (serpin family E member 2), whereas age‐induced genes included *Gdf15* (growth differentiation factor 15), *Cdkn2b* (cyclin‐dependent kinase inhibitor 2B) (Noh et al. 2026), *Osm* (oncostatin M) (La Belle et al. 2017), and *Akt1* (AKT serine/threonine kinase 1) (Li et al. 2026). Among these splenic candidates, *Txn and Gdf15* lacked age‐dependent expression changes in mice, while *Osm* and *Akt1* showed age‐related trends compared with their human and tree shrew orthologs. Moreover, *Pcna*, *Gclm*, and *Cdkn2b* displayed divergent age‐dependent expression profiles in mice versus humans and tree shrews (Figure S5C,D). Collectively, Mfuzz clustering and chord‐diagram analyses of liver and spleen transcriptomes demonstrate that tree shrews recapitulate many human‐like aging‐associated transcriptional features, especially for mid‐life inverted V‐shaped gene modules. By contrast, mice exhibit distinct gene‐set sizes and divergent age‐dependent expression patterns for multiple key aging‐related hepatic and splenic genes, highlighting that tree shrews serve as a complementary aging model to mice.

### 2.5 Effect of Senolytic Treatment on Functional and Transcriptomic Features in the Liver

Dasatinib and quercetin (D + Q) have been demonstrated to selectively clear senescent cells and improve physical functions in aged animals (Saccon et al. 2021; Takaya and Kishi 2024). To assess whether tree shrews respond to this anti‐aging intervention, we administered D + Q via oral gavage to aged animals and assessed physiological‐functional phenotypes together with transcriptomic alterations (Figure 5A). Although we did not observe significant improvement in circulating organ‐function biomarkers after 3‐month D + Q treatment (Figure S6A–Y), transcriptomic analysis revealed marked amelioration of aging markers at the tissue level.

**FIGURE 5:**
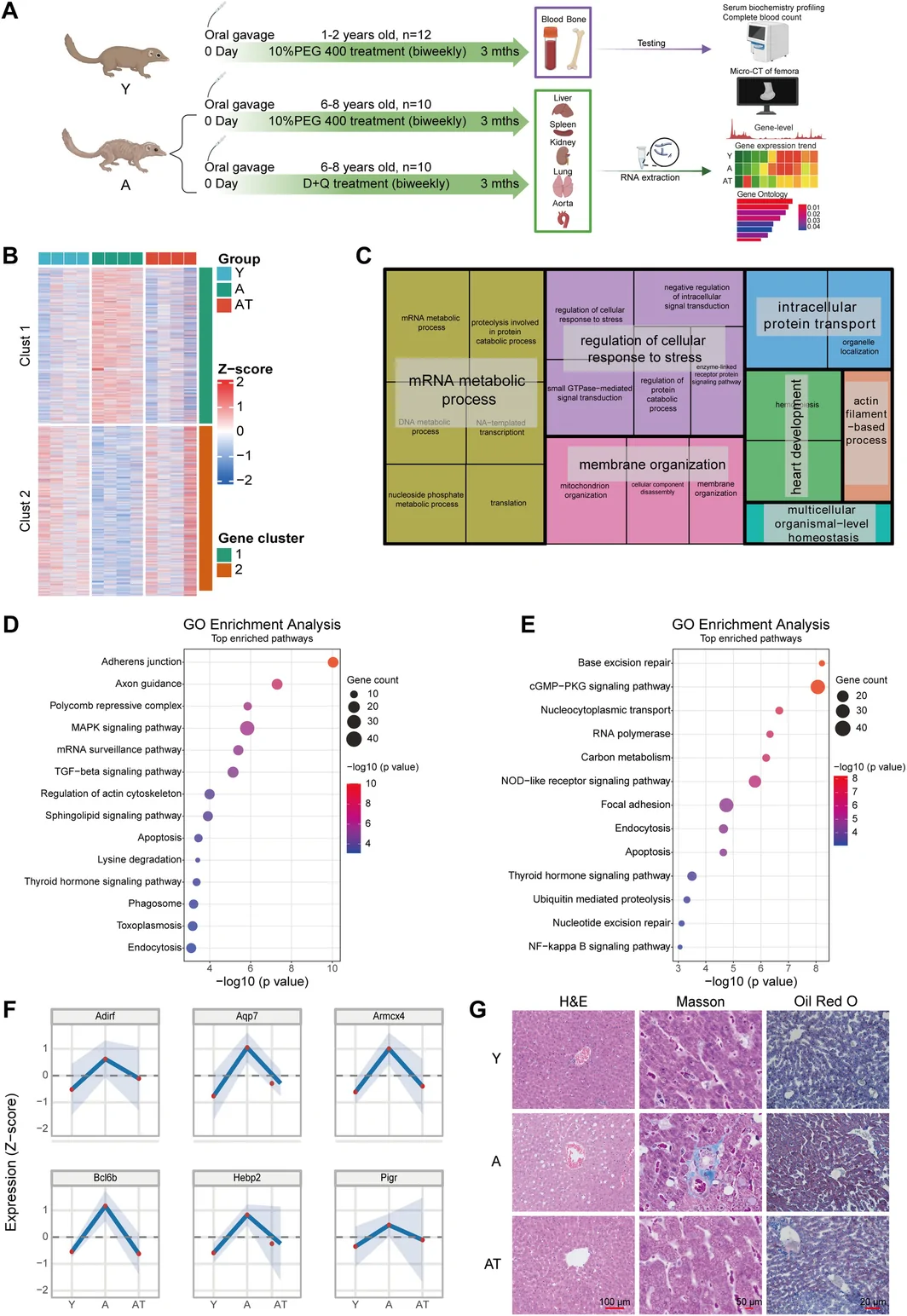
D + Q treatment ameliorates hepatic senescence in tree shrews. (A) Experimental design timeline for dasatinib and quercetin (D + Q) intervention. (B) Heatmap of differentially expressed genes (DEGs) across three experimental groups: Young controls (Y), aged controls (A), and D + Q‐treated aged tree shrews (AT). (C) REVIGO treemap visualization of GO Biological Process (BP) enrichment results. (D) GO enrichment analysis of downregulated DEGs. (E) GO enrichment analysis of upregulated DEGs. (F) Expression changes of senescence‐associated genes downregulated upon D + Q treatment (young, *n* = 4; aged, *n* = 4; AT, *n* = 4). (G) Representative H&E, Masson's trichrome staining, and Oil Red O staining of liver sections from young, aged controls, and D + Q‐treated aged tree shrews. Figure 5A was created with BioRender.com.

In liver tissue, Mfuzz clustering was performed to capture gene‐expression dynamics across young, aged, and D + Q‐treated aged groups. The corresponding heatmap illustrated profound transcriptional reprogramming triggered by D + Q intervention (Figure 5B). Functional enrichment of treatment‐responsive genes revealed that D + Q‐responsive expression patterns were primarily associated with pathways regulating mRNA metabolic processes, cellular response to stress, membrane organization, intracellular protein transport, heart development, and multicellular organismal‐level homeostasis, as identified by REduce and VIsualize Gene Ontology (REVIGO)‐based Gene Ontology summarization (Figure 5C). According to GO enrichment analysis, D + Q downregulated steatosis‐related signaling pathways (thyroid hormone signaling, sphingolipid signaling, lysine degradation, regulation of actin cytoskeleton, and endocytosis) and profibrotic pathways (TGF‐beta signaling pathway and MAPK signaling pathway) (Figure 5D). Protective pathways, including DNA repair and inflammation modulation, were enriched after treatment with D + Q (Figure 5E). Crucially, D + Q concurrently downregulated key senescence‐promoting genes, *Adirf*, *Aqp7*, *Armcx4*, *Bcl6b*, *Hebp2*, and *Pigr* (Figure 5F). These transcriptional signatures were consistent with histological alleviation of hepatic steatosis and fibroplasia following D + Q treatment (Figure 5G).

### 2.6 Transcriptomic Remodeling in Aged Aorta, Kidney, Lung, and Spleen in Response to Senolytic Treatment

To evaluate organ‐wide transcriptional responses to D + Q in aged tree shrews, we analyzed convergent transcriptomic alterations across the aorta, kidney, lung, and spleen. Integrative clustering resolved two core gene clusters: Cluster 1 genes were upregulated upon aging and repressed following D + Q intervention, whereas Cluster 2 genes were suppressed with age and restored after D + Q treatment (Figure 6A). Among the four examined organs, the aorta contained the largest number of D + Q‐responsive DEGs, highlighting it as a primary tissue undergoing D + Q‐driven transcriptional reprogramming (Figure 6B). Genes within Cluster 1 were enriched for myeloid cell development, endocytosis, epigenetic regulation of gene expression, positive regulation of autophagy, protein localization to the nucleus, mitochondrial organization, as well as cellular responses to radiation and growth stimuli. Genes in Cluster 2 were enriched in biological processes such as regulation of cell cycle phase transition, cellular response to oxidative stress, tRNA processing, double‐strand break repair, synaptic vesicle localization, and inner mitochondrial membrane organization, reflecting activation of cellular repair and homeostatic programs (Figure 6C). Several age‐elevated pro‐senescent genes, including inflammatory signaling kinase (inhibitor of nuclear factor kappa B kinase subunit beta, *Ikbkb*), immune transcription factor (signal transducer and activator of transcription 6, *Stat6*), and regulator of p53 activity (TP53 regulating kinase, *Tp53rk*), were downregulated by D + Q treatment. Conversely, D + Q activated X‐ray repair cross‐complementing protein 4 (*Xrcc4*), tubulin gamma complex component 2 (*Tubgcp2*), and HUS1 checkpoint clamp component (*Hus1*) (Figure 6D). Collectively, these data indicate that D + Q remodels aged tissue transcriptome by repressing pro‐inflammatory and epigenetic networks while reinforcing genome‐stability programs.

**FIGURE 6:**
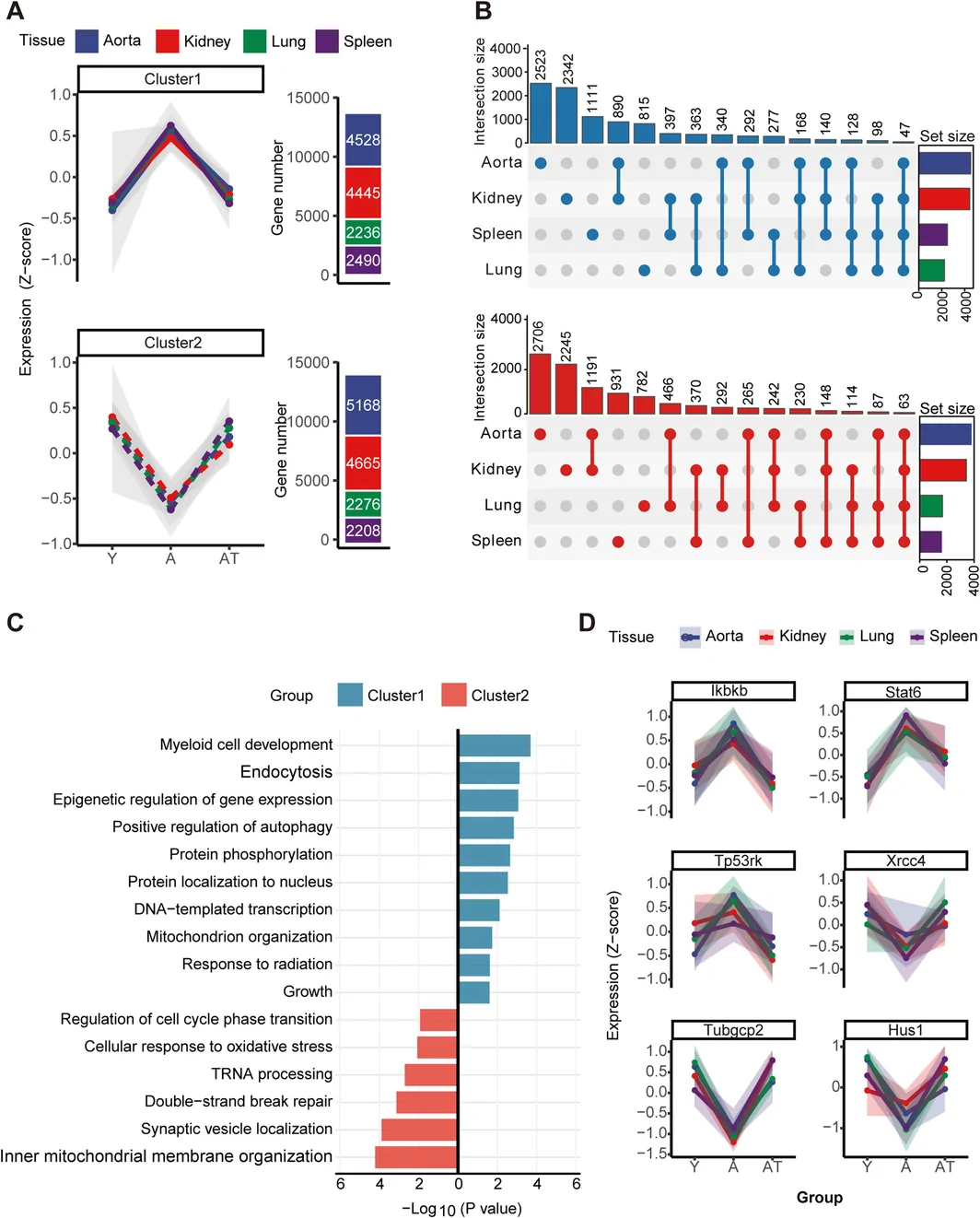
Multi‐tissue senescence pathway reprogramming by D + Q treatment in aged tree shrews. (A) Cluster 1 (top) comprises genes downregulated in aged tree shrews upon D + Q treatment; Cluster 2 (bottom) comprises genes upregulated following D + Q intervention. (B) UpSet plot quantifying tissue‐specific gene contributions for Cluster 1 (top panel) and Cluster 2 (bottom panel). (C) GO enrichment analysis of biological pathways enriched within Cluster 1 and Cluster 2 gene sets. (D) Expression dynamics of aging‐related genes in aged tree shrews after D + Q treatment.

We next assessed sex‐ and tissue‐specific transcriptional responses to D + Q and observed that female tree shrews exhibited more pronounced transcriptomic alterations following senolytic intervention. In the aorta, hierarchical clustering clearly separated young, aged, and D + Q‐treated aged groups (Figure S7A), reflecting robust age‐dependent transcriptional remodeling. Volcano plot analysis revealed significant upregulation of inflammatory and extracellular matrix (ECM) remodeling genes in aged female tree shrews relative to young female counterparts (Figure S7B), with enrichment in cytokine signaling and vascular fibrosis (Figure S7D). Notably, three‐month D + Q treatment reversed a large fraction of these age‐associated transcriptional changes, particularly genes linked to immune activation, fibrosis, and metabolic dysregulation (Figure S7C,E). Similar age‐driven transcriptomic divergence was observed in female tree shrew kidneys (Figure S8A). Aged kidneys displayed elevated expression of SASP‐related and oxidative stress response genes (Figure S8B), and KEGG enrichment highlighted perturbations in inflammation, cell adhesion, and metabolic dysfunction (Figure S8D). D + Q treatment dampened these pro‐aging pathways (Figure S8C,E), supporting the restoration of transcriptional homeostasis. In female lungs, aging was accompanied by transcriptomic signatures of chronic inflammation and tissue remodeling (Figure S8F,G,I), many of which were mitigated by D + Q treatment (Figure S8H,J).

To evaluate whether tree shrews represent a more suitable near‐human aging model, we first compared D + Q‐responsive differentially expressed genes (DEGs) between tree shrews and published mouse datasets (Figure S9A) (Hou et al. 2026). In the kidney, liver, and spleen, tree shrews consistently produced more D + Q‐responsive DEGs than mice (e.g., 641 up/292 down vs. 188/162 in the spleen), whereas the lung showed comparable numbers (313/224 vs. 361/65), indicating a broader overall D + Q transcriptional response in tree shrews.

We next performed UpSet‐plot analysis to intersect D + Q‐responsive DEGs with human aging‐associated gene signatures (Human‐Aged, age‐upregulated; Human‐Young, age‐downregulated). In both tree shrews and mice, the number of D + Q‐responsive genes overlapping with human aging‐associated signatures was limited. Nevertheless, in multiple tissues, tree shrews still exhibited a greater number of human aging‐associated genes, including genes whose human orthologs were age‐upregulated and suppressed by D + Q (e.g., 48 vs. 7 in the spleen; 32 vs. 7 in the lung) and genes whose human orthologs were age‐downregulated and restored by D + Q (e.g., 165 vs. 30 in the spleen; 76 vs. 9 in the liver) (Figure S9B)‐indicating that D + Q responses in tree shrews are more concordant with human aging regulation overall. We further conducted Gene Ontology Biological Process (GO‐BP) enrichment analysis separately on the two sets of intersecting genes across the four tissues: genes that were both age‐upregulated in humans and downregulated by D + Q in tree shrews (i.e., human aging‐up genes suppressed by D + Q), and genes that were both age‐downregulated in humans and upregulated by D + Q in tree shrews (i.e., human youthful genes restored by D + Q). Both gene sets were significantly enriched for aging‐relevant pathways, including cell–cell signaling, chronic inflammatory response, DNA repair, and DNA metabolic processes (Figure S9C), revealing core biological pathways remodeled by D + Q intervention.

Collectively, these transcriptomic data demonstrate that tree shrews recapitulate key molecular features of mammalian organ aging and respond effectively to senolytic treatment, reinforcing their utility as a translational pre‐clinical aging model.

## 3 Discussion

In this study, we present comprehensive molecular, physiological, and histological evidence establishing the tree shrew as a feasible alternative model for aging and age‐related disease research. Beyond close phylogenetic affinity to primates, this species recapitulates core mammalian aging hallmarks, including cellular senescence, tissue degeneration, systemic inflammation, and age‐dependent transcriptional reprogramming. Our findings support broader adoption of tree shrews for aging mechanistic studies and therapeutic pre‐clinical testing.

Mice have long dominated aging research owing to their short lifespan and amenability to genetic manipulation (Vanhooren and Libert 2013). Nevertheless, their substantial evolutionary divergence from humans limits translational extrapolations, particularly for human‐specific physiological and immune traits (Lu et al. 2021). As a primate‐related intermediate between rodents and NHPs, tree shrews offer key practical benefits: small body size, cost‐effective husbandry, prolific breeding, and fewer ethical and conservation‐related constraints (Xiao et al. 2017; Yao 2017; Yao et al. 2024). Tree shrew fibroblasts develop bona fide senescence upon irradiation, chemotherapy, or replicative exhaustion, with SA‐β‐gal positivity and elevated SASP production (Figure 1D–H; Figure S1C–E). Transcriptomics reveals conserved gene and pathway responses between irradiated tree shrew and human fibroblasts, including perturbed insulin‐PI3K/MAPK‐FOXO signaling and impaired proteostasis post‐IR (Figure S1H) (Ding et al. 2025; Fritsche et al. 2008; Zhang et al. 2011). Collectively, these data validate tree shrew fibroblasts as a suitable system for senescence research.

In vivo, aged tree shrews recapitulate key aging phenotypes including physical frailty, age‐associated bone loss, and increased hepatic tumor incidence (Figure 2). Senescent‐cell‐derived SASP drives inflammaging (Hao et al. 2026), and multi‐organ profiling demonstrates widespread upregulation of SASP‐related genes in aged tree shrews (Figure 3A–G). Notably, *Mmp9* is elevated across most organs except the liver (Figure 3H). MMP‐9, secreted by monocytes, macrophages, and neutrophils, is upregulated in aged mouse aortas (Zhang et al. 2023) and linked to NF‐κB signaling. Its interaction with TGF‐β promotes adipose tissue fibrosis and cancer proliferation (Yu et al. 2024), suggesting that MMP‐9 orchestrates aging cascades and may serve as a multi‐organ senescence marker in tree shrews.

Our RNA‐seq revealed widespread transcriptomic remodeling in aged tree shrew liver, spleen, aorta, kidney, and lung, with upregulation of ECM remodeling, cytokine‐immune signaling, oxidative stress, and metabolic dysfunction programs. A number of oxidative stress and senescence‐associated genes display concordant age‐dependent expression trajectories in the liver and spleen across humans and tree shrews. Nevertheless, a subset of these genes, such as *Hmox1*, *Prkaa1*, *Akt1*, and *Gdf15*, show opposite or nonsignificant age‐related expression changes in mice relative to humans and tree shrews, supporting the translational utility of tree shrews for aging research. Importantly, D + Q senolytic treatment reverses many of these transcriptomic changes, suppressing profibrotic pathways (TGF‐β, MAPK) and cancer‐related genes *Adirf* and *Aqp7* (Figure 5D,F) (Dai et al. 2020; Liu et al. 2024), accompanied by histological improvements in hepatic steatosis and fibroplasia (Figure 5G). These observations indicate that D + Q confers molecular protection by reshaping metabolic, profibrotic, and genome‐maintenance pathways, with the aorta showing the most robust transcriptomic response (Figure 6A,B). To further benchmark translational relevance, we intersected D + Q‐responsive DEGs with human aging gene signatures and compared overlaps with published mouse D + Q datasets. Across kidney, liver, lung, and spleen, D + Q‐regulated transcripts in tree shrews overlapped more extensively with human aging signatures than mouse counterparts. These results reinforce that tree shrews mount more human‐like transcriptional responses to senolytic intervention. Intriguingly, despite robust transcriptomic reversal upon senolytic treatment, we did not observe significant improvements in serum functional biomarkers (ALT, AST, CRE, etc.) or bone microstructure (Figure S6). This disconnection implies that transcriptomic changes may represent an early and sensitive molecular response to senolytic treatment, whereas the restoration of tissue architecture and organ function requires a substantially longer timeframe (Gerling et al. 2003). Collectively, these findings support tree shrews as a valuable model for longevity interventions, while underscoring the need for longer therapeutic windows or adjunctive therapies to translate molecular benefits into functional recovery.

Although the results are promising, several limitations should be noted. First, despite the available tree shrew genome sequence, its annotation is less complete compared with mice, and specialized analytical tools remain limited, hindering in‐depth molecular analyses. Second, the scarcity of standardized reagents, such as validated antibodies and enzymes, may introduce bias or assay failures in organ‐function measurements. Third, the absence of established transgenic lines precludes mechanistic studies, and well‐validated ethological assessment protocols are not yet available, restricting behavioral analyses. Collectively, these constraints inherently limit sample sizes and complicate experimental grouping in tree shrew studies.

In conclusion, we provide multi‐modal evidence supporting tree shrews as a translationally valuable model for aging and age‐related disease studies. Phylogenetic proximity to primates, human‐like aging phenotypes, and response to senolytic therapy make tree shrews a compelling alternative to conventional rodent models. This system may help bridge pre‐clinical discovery and clinical translation to advance geroscience research.

## 4 Experimental Section

### 4.1 Cell Isolation and Culture

TSDF and MDF were isolated from the ear tissue of tree shrews or C57BL/6 mice. All animal experimental protocols were approved by the Institutional Animal Care and Use Committee of Kunming Institute of Zoology, Chinese Academy of Sciences (Approval no. IACUC‐TE‐2024‐08‐004 and IACUC‐RE‐2024‐08‐012). Tissues were initially disinfected by immersion in 75% ethanol for 10 s, followed by thorough washing in phosphate‐buffered saline (PBS, B540627‐0500, Sangon Biotechnology, Shanghai, China) containing 10% penicillin–streptomycin (PS, 15140122, Gibco, MD, USA), and subsequently in PBS with 1% PS. After removing hair from the skin, the tissues were minced into ~1 mm^3^ fragments and transferred to centrifuge tubes. Pre‐warmed 0.25% trypsin (C25200072, Gibco, MD, USA) was added, and the tubes were sealed with parafilm and incubated at 37°C for 10 min. Digestion was halted by adding DMEM (C11995500BT, Gibco, MD, USA) supplemented with 10% fetal bovine serum (FBS, RYS‐F22‐05, Lanzhou Roya Bio‐Technology, Gansu, China). The suspension was centrifuged at 1000 rpm for 3 min, and the supernatant was discarded. The remaining tissue fragments were resuspended in 0.2% Collagenase Type II (2275MG100, Biofroxx, Hesse, Germany) and digested for 2 h in a shaking incubator.

Following digestion, the cell suspension was centrifuged at 800 rpm for 5 min. The pellet was washed in PBS containing 1% PS and filtered through a 200‐mesh cell strainer. After a final centrifugation, the cells were resuspended in DMEM supplemented with 20% FBS and 1% PS, then seeded into T25 culture flasks pre‐incubated with the same medium. TSELF was obtained from the Kunming Cell Bank of Type Culture Collection (KCB 2010134L, Yunnan, China). HDF was obtained as previously described (Hu et al. 2023). All cultures were maintained at 37°C in a 5% CO_2_ humidified incubator.

### 4.2 Senescence Induction

Senescence was induced in HDF, TSDF, TSELF, and MDF cells at ~70% confluency using two methods. To generate irradiation‐induced senescent cells (IR‐SnC), fibroblasts were exposed to 15 Gy X‐ray radiation using a small animal radiation research platform (Xstrahl Inc., Camberley, UK), following established protocols (Hu et al. 2023). For doxorubicin (Dox)‐induced senescence, cells were treated with 250 nM Dox (HY‐15142, MedChemExpress, Shanghai, China) in complete medium for 48 h, followed by culture in DMEM with 10% FBS. For replicative senescence, fibroblasts were continuously passaged for more than 20 passages until stable senescent phenotypes emerged.

### 4.3 SA‐β‐Galactosidase Staining

Senescence‐associated β‐galactosidase (SA‐β‐gal) activity was assessed using the Senescence β‐galactosidase Staining Kit (C0602, Beyotime, Shanghai, China), according to the manufacturer's instructions. Briefly, adherent cells were washed once with PBS, fixed in the provided fixative for 15 min at room temperature, and washed three times with PBS. Cells were then incubated with the β‐galactosidase staining solution at 37°C overnight in a CO_2_‐free dry incubator. Staining results were visualized and imaged under bright‐field microscopy on the following day.

### 4.4 qRT‐PCR

Total RNA was extracted using TRIzol reagent (15596018CN, Invitrogen, CA, USA), following the manufacturer's protocol. Reverse transcription was performed using HP All‐in‐One qRT Master Mix II (RT203, YUNBIO, Yunnan, China). The qRT‐PCR was conducted using 2 × universal SYBR qPCR master mix (QP101, YUNBIO, Yunnan, China). Relative gene expression was normalized to *GAPDH* and analyzed using the 2^−ΔΔCt^ method. Primer sequences are listed in Table S1.

### 4.5 Animals

Forty‐four healthy adult tree shrews were obtained from the laboratory animal center of the Kunming Institute of Zoology, Chinese Academy of Sciences. Animals were assigned into four age‐matched groups: young (1–2 years, *n* = 12), middle‐aged (3–4 years, *n* = 12), aged (6–8 years, *n* = 10), and D + Q treated aged tree shrews (6–8 years, *n* = 10). Aged tree shrews in the treatment group received oral gavage of 5 mg/kg dasatinib (HY‐10181, MedChemExpress, Shanghai, China) and 50 mg/kg quercetin (HY‐18085, MedChemExpress, Shanghai, China) twice weekly for 3 months. Control animals received an equivalent volume of 10% PEG400 vehicle (HY‐Y0873A, MedChemExpress, Shanghai, China). All animals were maintained under standard conditions (16°C–28°C, 40%–70% humidity, 12h light/dark cycle) with access to a standard diet and distilled water.

### 4.6 Tree Shrew Clinical Frailty Index Assessment

Frailty was assessed using a modified frailty index adapted from a previously described protocol (Whitehead et al. 2014). Health deficits were scored on a three‐point scale: 0 (absent), 0.5 (mild), and 1 (severe).

#### 4.6.1 Blood Biochemistry

After 12 h of fasting, tree shrews were anesthetized, and blood samples were collected via exsanguination. Blood samples were divided into two aliquots: one allowed to clot at room temperature for serum collection, and another collected in EDTA tubes. All samples were transported to the National Resource Center for Non‐Human Primates at the Kunming Institute of Zoology for biochemical analysis.

#### 4.6.2 Micro‐CT Analysis

Following sacrifice, the femora were fixed in a 10% formalin solution and then subjected to high‐resolution in vivo and in vitro Micro‐CT (NEMO II, PINGSENG Healthcare Inc., China). Three‐dimensional (3D) images were reconstructed and analyzed using Avatar v3.0 software to obtain quantitative parameters of both trabecular and cortical bone.

#### 4.6.3 H&E Staining

Tissues were fixed in 10% formalin, dehydrated through a graded ethanol series, cleared in xylene, and embedded in paraffin. Sections were cut using a microtome, mounted on slides, deparaffinized, rehydrated, stained with hematoxylin for 5–10 min, differentiated in acid alcohol, blued under running tap water, and counterstained with eosin for 1–3 min. Slides were dehydrated, cleared, and mounted with coverslips.

#### 4.6.4 Masson's Trichrome Staining

Deparaffinized and rehydrated sections were stained using the Masson's Trichrome Staining Kit (BA4079B, Baso, Guangdong, China), following the manufacturer's protocol.

#### 4.6.5 Oil Red O Staining

Liver tissues were fixed in 10% formalin, washed with PBS, and sequentially dehydrated with 20% and 30% sucrose. The dehydrated tissues were embedded in optimal cutting temperature compound (4583, SAKURA, CA, USA) and sectioned into 6‐μm‐thick slices using a cryostat. Subsequently, Oil Red O staining was performed according to the manufacturer's protocol (C0158M, Beyotime, Shanghai, China).

### 4.7 RNA‐Seq Data Processing

Raw RNA‐seq reads were processed using Trim Galore (v2.6) for adapter trimming and quality filtering. Clean reads were aligned to the tree shrew reference genome (TupChi_1.0) using the STAR aligner (v2.7.11a) with default settings. Read counting was performed using featureCounts (v2.0.6) from the Subread package.

#### 4.7.1 Differential Gene Expression Analysis

Read counts from both human and tree shrew samples were processed using DESeq2 (v1.42.1). Low‐count genes were filtered. Genes with an adjusted *p*‐value < 0.05 and |log2 fold change| > 1 were considered significantly up‐ or downregulated. For the cross‐species D + Q response comparison, since the mouse D + Q data were derived from a published dataset (Hou et al. 2026) whose original analysis used nominal *p*‐values, the same threshold was applied uniformly to the tree shrew and mouse datasets (|log2 fold change| ≥ 1 and *p* < 0.05) to ensure comparability. Hierarchical clustering was performed using heatmap.2 in R. Volcano plots were generated to visualize DEG patterns. Tissue‐specific DEGs were analyzed using ShinyGo for Kyoto Encyclopedia of Genes and Genomes (KEGG) pathway enrichment.

#### 4.7.2 Gene Set Score Analysis

Pathway activity was evaluated using GSVA (v1.50.5) with the gene set from MSigDB. Gene sets were transformed into per‐sample enrichment scores using the GSVA method with a Poisson kernel.

#### 4.7.3 Gene Clustering Analysis

Gene expression clustering was performed using the Mfuzz R package. Average expression values per group were standardized, and genes with similar expression trajectories across age groups were clustered; similar clusters were merged for interpretability. Cluster visualizations were generated using ggplot2 and pheatmap.

#### 4.7.4 WGCNA

Gene co‐expression networks were constructed using the WGCNA R package. Highly variable genes were selected, and hierarchical clustering was used to identify outliers. Soft‐thresholding power was determined by scale‐free topology criteria. Signed networks were generated, and modules were identified based on topological overlap. Module‐trait relationships were analyzed, and age‐associated modules were compared to Mfuzz clusters to identify shared genes for further investigation.

#### 4.7.5 UpSet Plot Visualization

The Complex Heatmap R package was used to generate UpSet plots to visualize overlaps among gene clusters across tissues. Intersections were computed and plotted based on shared gene sets.

#### 4.7.6 GO Enrichment Analysis

GO enrichment analysis was performed using the Metascape web platform (https://metascape.org). Analyses were conducted using default parameters, and the top enriched GO terms were visualized using ggplot2. To reduce redundancy and highlight key biological themes, we further summarized the GO enrichment results using REVIGO (http://revigo.irb.hr), a web‐based tool that clusters semantically similar GO terms and selects representative terms based on semantic similarity metrics.

### 4.8 Statistical Analysis

All data were analyzed using GraphPad Prism v10.4.1. Results are presented as mean ± standard error of the mean (SEM). Statistical comparisons between two groups were performed using Student's *t*‐test, and multiple group comparisons were assessed using one‐way ANOVA or the Kruskal–Wallis test, depending on data normality. A *p*‐value < 0.05 was considered statistically significant.

## Author Contributions

Qiuni Luo: methodology, validation, formal analysis, writing – original draft, investigation. Wenxian Xiao: investigation, validation, visualization, data curation. Xin Dong: software, formal analysis, data curation, writing – original draft. Ayesha Nisar: writing – original draft. Yuhua Ma: resources. Xiang Lai: validation. Raoxian Bai: methodology. Meiting Zi: project administration. Qi Geng: methodology. Qingyu Zhang: methodology. Cheng Zhang: investigation. Qiong Wang: methodology. Naheemat Modupeola Gold: investigation. Zhen Zhou: methodology. Li Hu: methodology. Ao Zhang: validation. Qinghua Zeng: validation. Jing Liu: resources. Sawar Khan: data curation, writing – review and editing. Longbao Lv: supervision, resources, funding acquisition. Yonghan He: conceptualization, writing‐review and editing, supervision, funding acquisition.

## Funding

This work was supported by the National Key R&D Program of China (2023YFC3603300 to Y.H.), Yunnan Fundamental Research Project (202602AS100006 to Y.H.), National Natural Science Foundation of China (82471599 and 8267055414 to Y.H.). Y.H. is supported by the Pioneer Hundred Talents Program of the Chinese Academy of Sciences and the Yunnan Revitalization Talent Support Program Young Talent Project.

## Conflicts of Interest

The authors declare no conflicts of interest.

## Supporting information


**Table S1:** Primers used for qRT‐PCR analysis.
**Figure S1:** Tree shrews represent a promising model for investigating cellular senescence. (A) Dentition comparison among rhesus macaques, tree shrews, and mice. (B) Claw structure comparison among rhesus macaques, tree shrews, and mice. (C) SA‐β‐gal staining of replicatively senescent cells (REP‐SnC); blue‐stained cells denote senescent cells. (D) Quantification of SA‐β‐gal‐positive cells in replicatively senescent TSDF relative to non‐SnC controls. (E) Quantitative RT‐PCR analysis of canonical senescence‐associated genes (*Cdkn1a*, *Cdkn2b*, *Il6*, *Glb1*, *Il1a*, *Tgfb2*, *Igfbp5*, *Mmp9*, *Mmp12*, *Ccl5*) in REP‐SnC versus non‐SnC TSDF. Data are shown as mean ± SEM. Statistical significance was determined using an unpaired two‐tailed Student's *t*‐test. *p < 0.05, **p < 0.01, ***p < 0.001, and ****p < 0.0001. (F) Volcano plots showing differentially expressed genes (DEGs) between IR‐treated and control young groups in HDF and TSDF. (G) Venn diagram depicting overlaps of DEGs between HDF and TSDF. (H) GSVA enrichment scores of senescence‐associated pathways in HDF and TSDF.
**Figure S2:** Age‐related phenotypic and biochemical alterations in tree shrews. (A) Representative age‐related phenotypes observed in aged tree shrews. (B‐M) Biochemical and hematological parameters across different age groups: (B) Aspartate aminotransferase (AST); (C) Alanine aminotransferase (ALT); (D) Creatinine (CRE); (E) Total bilirubin (TBIL); (F) Glucose (GLU); (G) Lactate dehydrogenase (LDH); (H) Red blood cell distribution width‐standard deviation (RDW‐SD); (I) Eosinophil percentage (Eos%); (J) Neutrophil percentage (Neu%); (K) Mean corpuscular hemoglobin concentration (MCHC); (L) Red blood cell distribution width‐coefficient of variation (RDW‐CV%); (M) Mean platelet volume (MPV). (N‐S) Representative H&E staining of tissues from aged tree shrews: (N) Kidney: red arrow marks lipofuscin accumulation; (O) Lung; (P) Spleen; (Q) Brown adipose tissue; (R) Ovary; (S) Quadriceps muscle: red arrow denotes internalized myonuclei.
**Figure S3:** RNA‐seq analysis of the liver and spleen in tree shrews. (A) Soft thresholding power selection for WGCNA in tree shrew liver tissue. (B) Soft thresholding power selection for WGCNA in tree shrew spleen tissue. (C) Hierarchical clustering dendrogram of co‐expressed genes in spleen tissues, partitioned into distinct modules by WGCNA. (D) Pearson correlation coefficients between module eigengenes and chronological age for each WGCNA module. (E) Scatterplot of module significance (*x*‐axis) versus gene significance (*y*‐axis) for the age‐correlated red module. (F) Mfuzz analysis clustering tree shrew genes into four distinct expression trends. (G) Venn diagram showing the overlap between age‐downregulated genes (Cluster 2) and red‐module member genes. (H) GO enrichment network for the intersecting genes, highlighting biological processes linked to cellular senescence and tissue aging.
**Figure S4:** Conserved age‐dependent gene expression trajectories in the liver across humans, tree shrews, and mice. (A) Four major aging‐related gene‐expression clusters for each species. (B) Chord‐diagram visualization of cross‐species homologous genes for each trajectory cluster, following the row order shown in panel A. (C, D) Representative expression profiles of aging‐associated genes. Genes shown in (C) are down‐regulated with aging in humans and tree shrews but exhibit different age‐dependent patterns in mice; genes in (D) are up‐regulated during aging in humans and tree shrews, whereas mice show different age‐dependent trajectories. Y, young; M, middle‐aged; A, aged. *N*, gene count per cluster.
**Figure S5:** Conserved age‐dependent gene expression trajectories in the spleen across humans, tree shrews, and mice. (A) Four major aging‐related gene‐expression clusters for each species. (B) Chord‐diagram visualization of cross‐species homologous genes for each trajectory cluster, following the row order shown in panel A. (C, D) Representative expression profiles of aging‐associated genes. Genes shown in (C) are down‐regulated with aging in humans and tree shrews but exhibit different age‐dependent patterns in mice; genes in (D) are up‐regulated during aging in humans and tree shrews, whereas mice show different age‐dependent trajectories. Y, young; M, middle‐aged; A, aged. *N*, gene count per cluster.
**Figure S6:** Effects of D + Q treatment on blood biochemistry, hematological parameters, and bone microstructure in aged tree shrews. (A–R) Serum biochemical and hematological indices in young (Y), aged (A), and D + Q treated aged (AT) tree shrews: (A) TP; (B) GLO; (C) GGT; (D) AST; (E) ALT; (F) CRE; (G) TBIL; (H) GLU; (I) LDH; (J) PLT; (K) PCT%; (L) Mon%; (M) RDW‐SD; (N) Eos%; (O) Neu%; (P) MCHC; (Q) RDW‐CV; (R) MPV. (S) Representative 3D reconstructed micro‐CT images of the distal femoral metaphysis from Y, A, and AT. Scale bar, 1 mm. (T–Y) Quantitative micro‐CT analysis of trabecular bone microarchitectural parameters: (T) Tb. BV/TV; (U) Tb. BS; (V) Tb. BS/TV; (W) Conn. D; (X) Tb. TMC; (Y) Tb. Pf.
**Figure S7:** RNA‐seq analysis of aortic tissues from female tree shrews. (A–E) Transcriptomic profiling of tree shrew aortic tissues. (A) Hierarchical clustering analysis of global gene expression profiles in young (Y), aged (A), and D + Q treated aged (AT) tree shrews; (B) Volcano plot illustrating DEGs in the A group versus the Y group; (C) Volcano plot illustrating DEGs in the AT group versus the A group; (D) KEGG pathway enrichment analysis for upregulated DEGs identified in the A vs. Y comparison; (E) KEGG pathway enrichment analysis for downregulated DEGs identified in the AT vs. A comparison.
**Figure S8:** RNA‐seq analysis of kidney and lung tissues from female tree shrews. (A–E) Transcriptomic profiling of kidney tissues. (A) Hierarchical clustering analysis of global gene expression profiles in young (Y), aged (A), and D + Q‐treated aged (AT) tree shrews; (B) Volcano plot showing DEGs in the A versus Y groups. (C) Volcano plot showing DEGs in the AT versus A groups. (D) KEGG pathway enrichment analysis of upregulated DEGs identified in the A vs. Y comparison. (E) KEGG pathway enrichment analysis for downregulated DEGs derived from the AT vs. A comparison. (F–J) Transcriptomic profiling of lung tissues. (F) Hierarchical clustering analysis of global gene expression profiles in young (Y), aged (A), and D + Q‐treated aged (AT) tree shrews; (G) Volcano plot showing DEGs in the A relative to the Y group. (H) Volcano plot showing DEGs in AT relative to the A group. (I) KEGG pathway enrichment analysis for upregulated DEGs derived from the A vs. Y comparison. (J) KEGG pathway enrichment analysis for downregulated DEGs derived from the AT vs. A comparison.
**Figure S9:** Tree shrews serve as a D + Q‐responsive model that more closely recapitulates human aging. (A) Number of DEGs upon D + Q treatment in four tissues (kidney, liver, lung, spleen) from mice and tree shrews. Red bars indicate D + Q‐upregulated genes; blue bars indicate D + Q‐downregulated genes. DEGs were defined as |log_2_FC| ≥ 1 and *p* < 0.05. (B) UpSet plots showing the overlap among D + Q‐responsive DEGs in mice and tree shrews, as well as human aging‐associated genes across four tissues. Dots denote the gene sets being compared, and vertical bars represent the gene count of each exact intersection. The blue line represents overlaps between human‐Young genes (genes declining with age) and genes up‐regulated by D + Q treatment in tree shrews. The red line represents overlaps between human‐Aged genes (genes increasing with age) and genes down‐regulated by D + Q treatment in tree shrews. (C) GO‐BP enrichment analyses for the blue‐ and red‐line gene sets in each tissue. Bubble plots display representative aging‐relevant pathways. Bubble size corresponds to the number of genes within each pathway, and bubble color indicates enrichment significance (−log10 *p*).

## Data Availability

Raw RNA‐seq data are deposited in GSA (NGDC, CAS) under accession PRJCA072680 (https://ngdc.cncb.ac.cn/gsa).
