# Supplementary material for: Tree Shrew as an Emerging Model for Aging Research: Characterization of Senescence and Therapeutic Interventions
Source: Aging Cell. 2026 Nov 3;25(11):e70781. doi: 10.1111/acel.70781 (PMC13731738; doi:10.1111/acel.70781)
Supplement: Supplementary file 1 [file ACEL-25-e70781-s001.docx]

| **Table S1. Primers used for qRT-PCR analysis.** | | | |
| --- | --- | --- | --- |
| **Name** | **Forward primer (5')** | | **Reverse primer (3')** |
| *Cdkn2b* | ATGCGTGAGGAGGACAAGA | GTTGACTCCGTTGGGATCTG | |
| *Cdkn1a* | GTGGAACTTCGACTTCGTCA | GCAAGTAGACCTTAGGCAGG | |
| *Il6* | TCCTAGTGATGGCTACTGCTCTTC | TTATCTGTGGAGGTGGGTTCTGG | |
| *Il1a* | TCAAGGAGAGCGTGGTAGTAACAG | TCTTCTACTGGATCACTGGCGATG | |
| *Ccl5* | CAACCCAGCAGTTGTGTTTATC | AGAGTTGATGTATTCCCGAACC | |
| *Igfbp5* | CCAACTGTGACCGCAAAGGATTC | TTGTCCACGCACCAGCAGAG | |
| *Mmp12* | GATCCCAGCACATTTCGCCTCTC | AGGGTCGCAGATAGTTGAGTCAGG | |
| *Cxcl10* | GCCAAGTGCCATTCTCCTTTTCTG | TGATGCACGTACAGCGTACAGTTC | |
| *Glb1* | GAATCTATTGTTCTTCGCTCTTCTGAC | GTGATAATTGGTCCTCCGTTCTGATAG | |
| *Tgfb2* | GCCTGAACAGCGGATCGAACTG | TGAAGCCACTCGTGAACAGCATC | |
| *Igfbp2* | CGGTCAGCGTGGAGAGTGC | GCCTCCTGTTGCTCATTGTAGAAG | |
| *Il1b* | GATGGATTACTACAGTGCCGAAGAAG | CTGGATGCCCTTGTCTGGAGAG | |
| *Gapdh* | CGCCAACATCAAATGGGGTG | ATGGTTCACGCCCATCACAA | |
| *Mmp9* | AGGATCCAGCCTGTTCCTTG | TTAGGGCCAGGACCGTAGA | |

**Figure S1. Tree shrews represent a promising model for investigating cellular senescence. (A)** Dentition comparison among rhesus macaques, tree shrews, and mice. **(B)** Claw structure comparison among rhesus macaques, tree shrews, and mice. **(C)** SA-β-gal staining of replicatively senescent cells (REP-SnC); blue-stained cells denote senescent cells. **(D)** Quantification of SA-β-gal-positive cells in replicatively inducedsenescent TSDF relative to non-SnC controls. **(E)** Quantitative RT-PCR analysis of canonical senescence-associated genes (*Cdkn1a*, *Cdkn2b*, *Il6*, *Glb1*, *Il1a*, *Tgfb2*, *Igfbp5*, *Mmp9*, *Mmp12*, *Ccl5*) in REP-SnC versus non-SnC TSDF. Data are shown as mean ± SEM. Statistical significance was determined using an unpaired two-tailed Student’s *t*-test. **p* < 0.05, ***p* < 0.01, ****p* < 0.001, and *****p* < 0.0001. **(F)** Volcano plots showing differentially expressed genes (DEGs) between IR-treated and control young groups in HDF and TSDF. **(G)** Venn diagram depicting overlaps of DEGs between HDF and TSDF. **(H)** GSVA enrichment scores of senescence-associated pathways in HDF and TSDF.

**Figure S2. Age-related phenotypic and biochemical alterations in tree shrews.** **(A)** Representative age-related phenotypes observed in aged tree shrews. **(B-M)** Biochemical and hematological parameters across different age groups: **(B)** Aspartate aminotransferase (AST); **(C)** Alanine aminotransferase **(**ALT); **(D)** Creatinine (CRE); **(E)** Total bilirubin (TBIL); **(F)** Glucose (GLU); **(G)** Lactate dehydrogenase (LDH); **(H)** Red blood cell distribution width-standard deviation (RDW-SD); **(I)** Eosinophil percentage (Eos%); **(J)** Neutrophil percentage (Neu%); **(K)** Mean corpuscular hemoglobin concentration (MCHC); **(L)** Red blood cell distribution width-coefficient of variation (RDW-CV%); **(M)** Mean platelet volume (MPV). **(N-S)** Representative H&E staining of tissues from aged tree shrews: **(N)** Kidney: red arrow marks lipofuscin accumulation; **(O)** Lung; **(P)** Spleen; **(Q)** Brown adipose tissue; **(R)** Ovary; **(S)** Quadriceps muscle: red arrow denotes internalized myonuclei.

**Figure S3. RNA-seq analysis of the liver and spleen in tree shrews. (A)** Soft thresholding power selection for WGCNA in tree shrew liver tissue. **(B)** Soft thresholding power selection for WGCNA in tree shrew spleen tissue. **(C)** Hierarchical clustering dendrogram of co-expressed genes in spleen tissues, partitioned into distinct modules by WGCNA. **(D)** Pearson correlation coefficients between module eigengenes and chronological age for each WGCNA module. **(E)** Scatterplot of module significance (x-axis) versus gene significance (y-axis) for the age-correlated red module. **(F)** Mfuzz analysis clustering tree shrew genes into four distinct expression trends. **(G)** Venn diagram showing the overlap between age‑downregulated genes (cluster 2) and red-module member genes. **(H)** GO enrichment network for the intersecting genes, highlighting biological processes linked to cellular senescence and tissue aging.

**Figure S4. Conserved age‑dependent gene expression trajectories in the liver across humans, tree shrews, and mice. (A)** Four major aging‑related gene‑expression clusters for each species. **(B)** Chord‑diagram visualization of cross‑species homologous genes for each trajectory cluster, following the row order shown in panel A. **(C‑D)** Representative expression profiles of aging‑associated genes. Genes shown in (C) are down‑regulated with aging in humans and tree shrews but exhibit different age‑dependent patterns in mice; genes in (D) are up‑regulated during aging in humans and tree shrews, whereas mice show different age‑dependent trajectories. Y, young; M, middle‑aged; A, aged. N, gene count per cluster.

**Figure S5. Conserved age-dependent gene expression trajectories in the spleen across humans, tree shrews, and mice. (A)** Four major aging‑related gene‑expression clusters for each species. **(B)** Chord‑diagram visualization of cross‑species homologous genes for each trajectory cluster, following the row order shown in panel A. **(C‑D)** Representative expression profiles of aging‑associated genes. Genes shown in (C) are down‑regulated with aging in humans and tree shrews but exhibit different age‑dependent patterns in mice; genes in (D) are up‑regulated during aging in humans and tree shrews, whereas mice show different age‑dependent trajectories. Y, young; M, middle‑aged; A, aged. N, gene count per cluster.

**Figure S6. Effects of D+Q treatment on blood biochemistry, hematological parameters, and bone microstructure in aged tree shrews. (A–R)** Serum biochemical and hematological indices in young (Y), aged (A), and D+Q treated aged (AT) tree shrews: **(A)** TP; **(B)** GLO; **(C)** GGT; **(D)** AST; **(E)** ALT; **(F)** CRE; **(G)** TBIL; **(H)** GLU; **(I)** LDH; **(J)** PLT; **(K)** PCT%; **(L)** Mon%; **(M)** RDW-SD; **(N)** Eos%; **(O)** Neu%; **(P)** MCHC; **(Q)** RDW-CV; **(R)** MPV. **(S)** Representative 3D reconstructed micro-CT images of the distal femoral metaphysis from Y, A, and AT. Scale bar, 1 mm. **(T-Y)** Quantitative micro-CT analysis of trabecular bone microarchitectural parameters: **(T)** Tb. BV/TV; **(U)** Tb. BS; **(V)** Tb. BS/TV; **(W)** Conn. D; **(X)** Tb. TMC; **(Y)** Tb. Pf.

**Figure S7. RNA-seq analysis of aortic tissues from female tree shrews.** **(A–E)** Transcriptomic profiling of tree shrew aortic tissues. **(A)** Hierarchical clustering analysis of global gene expression profiles in young (Y), aged (A), and D+Q treated aged (AT) tree shrews; **(B)** Volcano plot illustrating DEGs in the A group versus the Y group; **(C)** Volcano plot illustrating DEGs in the AT group versus the A group; **(D)** KEGG pathway enrichment analysis for upregulated DEGs identified in the A vs. Y comparison; **(E)** KEGG pathway enrichment analysis for downregulated DEGs identified in the AT vs. A comparison.

**Figure S8. RNA-seq analysis of kidney and lung tissues from female tree shrews. (A–E)** Transcriptomic profiling of kidney tissues. **(A)** Hierarchical clustering analysis of global gene expression profiles in young (Y), aged (A), and D+Q-treated aged (AT) tree shrews; **(B)** Volcano plot showing DEGs in the A versus Y groups. **(C)** Volcano plot showing DEGs in the AT versus A groups. **(D)** KEGG pathway enrichment analysis of upregulated DEGs identified in the A vs. Y comparison. **(E)** KEGG pathway enrichment analysis for downregulated DEGs derived from the AT vs. A comparison. **(F-J)** Transcriptomic profiling of lung tissues. **(F)** Hierarchical clustering analysis of global gene expression profiles in young (Y), aged (A), and D+Q-treated aged (AT) tree shrews; **(****G)** Volcano plot showing DEGs in the A relative to the Y group. **(H)** Volcano plot showing DEGs in AT relative to the A group. **(I)** KEGG pathway enrichment analysis for upregulated DEGs derived from the A vs. Y comparison. **(J)** KEGG pathway enrichment analysis for downregulated DEGs derived from the AT vs. A comparison.

**Figure S9. Tree shrews serve as a D+Q‑responsive model that more closely recapitulates human aging. (A)** Number of DEGs upon D+Q treatment in four tissues (kidney, liver, lung, spleen) from mice and tree shrews. Red bars indicate D+Q‑upregulated genes; blue bars indicate D+Q‑downregulated genes. DEGs were defined as |log_2_FC| ≥ 1 and *p* < 0.05. **(B)** UpSet plots showing the overlap among D+Q‑responsive DEGs in mice and tree shrews, as well as human aging‑associated genes across four tissues. Dots denote the gene sets being compared, and vertical bars represent the gene count of each exact intersection. The blue line represents overlaps between human-Young genes (genes declining with age) and genes up-regulated by D+Q treatment in tree shrews. The red line represents overlaps between human-Aged genes (genes increasing with age) and genes down-regulated by D+Q treatment in tree shrews. **(C)** GO‑BP enrichment analyses for the blue‑ and red‑line gene sets in each tissue. Bubble plots display representative aging‑relevant pathways. Bubble size corresponds to the number of genes within each pathway, and bubble color indicates enrichment significance (−log10 *P*).
